# Supplementary material for: Comparative genomics provides new insights into the diversity, physiology, and sexuality of the only industrially exploited tremellomycete: Phaffia rhodozyma
Source: BMC Genomics. 2016 Nov 9;17:901. doi: 10.1186/s12864-016-3244-7 (PMC5103461; doi:10.1186/s12864-016-3244-7)
Supplement: Additional file 6: — List of orphan genes with links to PFAM (related to Additional file 1: Table S1). (ZIP 1428 kb) [file 12864_2016_3244_MOESM6_ESM.zip › PFAM_UNIQUE/G00129_P.html]

#                                                                            --- full sequence --- -------------- this domain -------------   hmm coord   ali coord   env coord
  
# target name        accession   tlen query name           accession   qlen   E-value  score  bias   #  of  c-Evalue  i-Evalue  score  bias  from    to  from    to  from    to  acc description of target
  
#------------------- ---------- ----- -------------------- ---------- ----- --------- ------ ----- --- --- --------- --------- ------ ----- ----- ----- ----- ----- ----- ----- ---- ---------------------
  
G00129\_P             -            815 DUF572               PF04502.8    324       4.1    5.2   7.6   1   1     0.064       8.5    4.1   7.6   180   277   157   264   126   306 0.41 - Family of unknown function (DUF572)  
G00129\_P             -            815 Ecl1                 PF12855.2     43     0.018   13.0   0.3   1   1   1.7e-05     0.035   12.1   0.3    19    38    44    63    32    68 0.77 - Life-span regulatory factor  
G00129\_P             -            815 FTO\_CTD              PF12934.2    172     0.042   12.1   1.7   1   1   1.2e-05     0.071   11.3   1.7    75   131   144   202   138   219 0.72 - FTO C-terminal domain  
G00129\_P             -            815 Peptidase\_S64        PF08192.6    695      0.71    6.8   9.0   1   1    0.0028       1.1    6.1   9.0    12   194   154   339   144   361 0.54 - Peptidase family S64  
G00129\_P             -            815 Snf7                 PF03357.16   171       1.9    6.5   5.4   1   1     0.009       3.9    5.5   5.4     8    61   146   196   140   208 0.83 - Snf7
